# Pigmented Fungiform Papillae (PFP) of the Tongue: A Systematic Review of Current Aetiopathogenesis and Pathophysiology

**DOI:** 10.3390/pathophysiology29030043

**Published:** 2022-09-09

**Authors:** Meircurius Dwi Condro Surboyo, Lakshman Samaranayake, Arvind Babu Rajendra Santosh, Nurina Febriyanti Ayuningtyas, Sisca Meida Wati, Retno Pudji Rahayu, Francisco Urbina, Winni Langgeng Kuntari, Sesaria Junita Mega Rahma Syahnia, Karlina Puspasari, Adiastuti Endah Parmadiati, Diah Savitri Ernawati

**Affiliations:** 1 Department of Oral Medicine, Faculty of Dental Medicine, Universitas Airlangga, Surabaya 60132, Indonesia; 2 Faculty of Dentistry, University of Hong Kong, Hong Kong, China; 3 School of Dentistry, Faculty of Medical Sciences, Mona Campus, The University of The West Indies, Kingston JMAAW14, Jamaica; 4 Department of Oral Pathology and Maxillofacial, Faculty of Dental Medicine, Universitas Airlangga, Surabaya 60132, Indonesia; 5 Dermatologist in Private Practice, Santiago de Chile 7580258, Chile; 6 Bachelor Dental Science Program, Faculty of Dental Medicine, Universitas Airlangga, Surabaya 60132, Indonesia; 7 Oral Medicine Specialist Program, Faculty of Dental Medicine, Universitas Airlangga, Surabaya 60132, Indonesia

**Keywords:** tongue, papillae, fungiform, pigmentation, melanocytes

## Abstract

The pigmentation of the fungiform papillae of the tongue is a rare idiopathic condition in which only the fungiform papillae appear hyperpigmented. In the absence of any reviews on the subject, we conducted a systematic review of the aetiopathogenesis and pathophysiology of pigmented fungiform papillae (PFP) of the tongue, including its demographic and histopathological features, trying to outline a possible aetiology. The preferred reporting items for systematic reviews and meta-analyses (PRISMA) was performed using PubMed, Scopus, EMBASE databases and manual searches, for publications between January 1974 and July 2022. Inclusion criteria were case reports defining patients’ characteristics, their general medical and dental conditions, histopathological and/or immunohistochemical findings, all with a final definitive diagnosis of PFP. Overall, 51 studies comprising 69 cases of PFP which included histopathological descriptions were reviewed. Prominent features consisted of hyperpigmentation of melanocytes, melanophages, chromatophores, and a lymphocytic infiltrate in the subepidermal area of the fungiform papillae. On special staining, PFP contained melanin, not iron or hemosiderin. On immunohistochemistry, immune-reactive CD3+ T lymphocytes, S-100 and Sox10, but non-immune-reactive melan-A intraepithelial melanocytes were noted in some studies. The presence of hyperpigmented melanocytes and melanophages, with non-immune-reactive melan-A, suggests that PFP are a benign and physiological form of pigmentation. The inflammatory infiltrates described in some papillary lesions could possibly be due to traumatic events during mastication. Nevertheless, the true reasons for the hyperpigmentation of the fungiform papillae are as of yet elusive, and remain to be determined.

## 1. Introduction

Oral mucosal pigmentation can be induced by either intrinsic or extrinsic causes [1]. The causes can be categorised as physiological [2], or pathological. The associated pathological causes include systemic infections such as the human immunodeficiency virus (HIV) infection [3,4,5,6,7,8], malignancies [9], inflammatory conditions [10,11], iatrogenic and drug-induced hyperpigmentations [12,13,14,15,16,17,18], tobacco-related [19], or idiopathic aetiology [20,21,22]. Overwhelmingly, most oral mucosal hyperpigmentations are innocuous and appear as a normal variant of the mucous colouration, which does not require further intervention (e.g., racial hyperpigmentation and amalgam tattoos), except when patients are deeply concerned.

Other well-recognised conditions that affect lingual mucosa are fissured tongue (also known as scrotal tongue or lingua plicata) [23,24], geographic tongue [25], coated tongue, hairy tongue [26], ankyloglossia [27], crenated tongue, and lingual varices [28]. Some rare conditions/syndromes, such as Laugier–Hunziker syndrome [3,29] and Dowling–Degos disease (DDS) [30], may present with PFP of the tongue.

**Figure 1:**
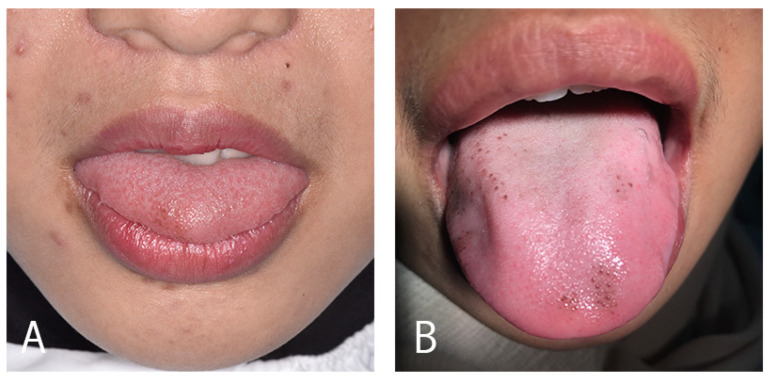
Clinical appearance of PFP, showing the Holzwanger Type 1 (**A**) and Type 2 (**B**) variants.

Fungiform papillae are located on the tip and lateral portions of the tongue; infrequently, they may become hyperpigmented solely, appearing as mushroom-like structures with a brown or dark colour [31] (Figure 1).

Clinically, PFP can be easily diagnosed by careful naked eye examination, with or without dermoscopy [32]. The latter provides a much clearer visualisation of the lesions, which usually have a cobblestone or rose-petal appearance [32]. Some authors have used tissue biopsy as a diagnostic tool to rule out underlying malignancies.

In the absence of any reviews on the subject in the literature to our knowledge, we conducted the current systematic review on PFP, its possible aetiopathogenesis, and pathophysiology.

## 2. Materials and Methods

A systematic search of the literature, including publications from January 1974 to July 2022, was conducted using the databases PubMed, Scopus, and EMBASE, as well as a manual search utilizing the MESH terms and keywords “pigmented fungiform papillae”, “oral”, and “tongue”.

The PFP have been classified into three types by Holzwanger [33] (Figure 1). In type 1, the pigmentation affects a well circumscribed area on the anterolateral sides or towards the tip of the tongue; in type 2, the pigmentation affects a few fungiform papillae of the dorsum of the tongue; while in type 3, the pigmentation involves all of the dorsum fungiform papillae.

From all databases, a total of 82 articles were identified, and those with duplicate titles/resources were excluded. The remaining total of 51 articles was then screened based on the availability of the abstracts and full texts. Further filtering of these yielded 51 articles for final review. The 51 articles were divided into two categories, 21 articles, encompassing 36 cases that presented or discussed a PFP case by including histopathological findings accompanied by another examination. The other 30 articles encompassed 33 cases that presented or discussed a PFP case.

## 3. Results

### 3.1. Cumulative Data

The review yielded 51 studies comprising 69 cases of PFP, and all of them were case reports. Of these, approximately three-quarters of the cases were reported in females (55/69; 79.71%), the youngest being 7 years old and the oldest 65 years old. Most patients were from Asia, Caucasian (*n* = 1), Indonesia (Javanese) (*n* = 2), Japanese (*n* = 3), Taiwanese (*n* = 1), Indian (*n* = 2), Korean (*n* = 3), and Vietnamese (*n* = 1) origin, while the remainder were Europeans (Hispanic; *n* = 3 and Italian; *n* = 2), African (*n* = 13), American (*n* = 4), or mixed ethnicity (*n* = 15) origin. The ethnicity or nationality was not reported in 15 cases (Table 1).

### 3.2. Clinical Appearance of PFP

PFP were almost equally distributed over the tongue, in the following decreasing order of frequency: dorsal (13/66); antero-lateral (11/69); anterial (9/69); lateral (3/66); antero-dorsal (1/69); antero-dorsal (1/69); latero-distal (1/69); both anterial and dorsal (3/69); anterial and lateral (1/69); anterial, lateral, and dorsal (2/69); dorsal and lateral (8/69); and antero-lateral and dorsal (1/69). In 16/69 cases lesion location was not mentioned (Table 1).

The dermoscopy findings reported were: cobblestone appearance (10/69), rose-petal appearance (17/69), both cobblestone and rose-petal appearance (2/69), and non-specific and unreported (39/69). The pigmentations were classified as either Type 1 or 2, as per the Holzwanger categorisation of the affected fungiform papillae [33] (Figure 1). Accordingly, 22 of 69 (31.9%) cases could be categorised as Type 1, 43 of 69 (62.3%) as Type 2, and 1 of 69 (1.5%) as Type 1 and Type 2. The remainder (2/29) were unclassifiable due to the absence of clinical photos (Table 1).

### 3.3. Associated Dermatological and Other Systemic Manifestations

Twenty-six studies reported associated skin and mucosal findings in addition to PFP, such as patch stage mycosis fungoides (1/69) [34], or IA mycosis fungoides (1/69) [35], acanthosis nigricans (1/69) [36], pigmented macules on trunk (1/69) [37] or lips (1/69) [38], hand eczema (1/69) [39], asymptomatic skin hyperpigmentation (1/36) [40], yellowish hyperpigmentation of sclera and conjunctival mucosa (1/36) [41], Fitzpatrick skin type II (1/69) [42], Fitzpatrick skin type III (13/69) [43,44,45], Fitzpatrick skin type IV (11/69) [46,47,48,49,50,51,52,53,54], Dowling–Degos disease (1/69) [30], and similar PFP affects also seen in another family member, especially the mother (2/69) [55,56]. A few studies clearly stated that skin, mucosal, or nail pigmentation was not found [31,33,46,49,50,52,53,54,57,58,59,60] or that parents or family members did not present similar pigmentation of the oral mucosa [38,61,62].

**Table 1:** Characteristics of PFP of the tongue as reported in selected studies.

Gender	Age	Ethnicity	Location of Affected Papillae	Clinical Appearance	Dermoscopy	Type	Ref.
Female	7	Ethiopian	Antero-dorsal	Multiple brown pigmented spots	RP	Type 2	[63]
Female	8	African	Antero-lateral	Multiple dark pinhead papules	RP	Type 2	[57]
Female	9	Japanese	Anterial and dorsal	Multiple pigmentation	CS	Type 1	[64]
Female	9	NR	Lateral	Multiple pigmentation	NR	Type 1	[43]
Female	10	Indian	Dorsal and lateral	Multiple sharply bordered macules	NR	Type 2	[55]
Female	12	Moroccan	Anterial	Multiple hyperpigmented papillae in a diffuse and symmetrical pattern	RP	Type 1	[46]
Female	12	African	Anterial, lateral, and dorsal	Multiple hyperpigmented papillae presenting as dark patches	RP	Type 2	[36]
Female	12	NR	Anterial and dorsal	Multiple discrete tan-brown pinhead papules	RP	Type 2	[58]
Female	12	NR	Dorsal and lateral	Brown pigmentations	RP CS	Type 1	[47]
Female	12	Asian	Anterial	Tiny pigmented macules	CS	Type 2	[65]
Female	13	South Asian	Anterial	Light to dark brown pigmentation, round or polygonal in shape, and circumscribed	CS	Type 1	[66]
Female	13	Mexican	NR	Multiple hyperchromic macules, light brown, mottled, 1 mm in diameter	RP	Type 2	[48]
Female	15	NR	Anterial, lateral, and dorsal	Asymptomatic and multiple brown pigmentations	CS RP	Type 1 Type 2	[49]
Female	15	NR	Dorsal	Multiple brown macules	NR	Type 2	[31]
Female	18	North African	Anterial	Multiple small erythematous and hyperpigmented papules	RP	Type 1	[50]
Female	18	Black	Dorsal	Bluish-black to black macular hyperpigmentation measuring 30–70 mm	NR	Type 1	[33]
Female	18	NR	Dorsal	Dark spots	NR	NCP	[51]
Female	20	NR	Antero-lateral and dorsal	Multiple brown macules	RP	Type 2	[67]
Female	20	Moroccan	Dorsal	NR	NR	Type 2	[68]
Female	21	NR	Antero-lateral	Irregularly distributed pigmentation	RP	Type 2	[42]
Female	23	NR	Antero-lateral	Multiple hyperpigmented papillae in a diffuse pattern	NR	Type 2	[56]
Female	24	Hispanic	Dorsal and lateral	Diffuse punctate pigmentation in a symmetrical pattern, and others grouped in a mottled pattern	RP	Type 1	[69]
Female	25	Saudi	Dorsal	Diffuse tan, brown, patches with prominent dark papillae	NR	Type 2	[52]
Female	25	Korean	Antero-lateral	Multiple dark brownish macules	NR	Type 1	[70]
Female	33	Korean	Antero-lateral	Multiple dark brownish macules	NR	Type 1	
Female	26	Black	Antero-lateral	Small reddish-brown pigmented lesions	NR	Type 1	[71]
Female	26	Indian	Dorsal	Multiple tiny brown macules	NR	Type 1	[37]
Female	27	Italian	Antero-lateral	Multiple blue-grey pigmentation, diffuse with a symmetrical pattern	RP	Type 2	[72]
Female	28	Haitian	Antero-lateral	Multiple dark brown macules	NR	Type 2	[73]
Female	28	NR	Dorsal	Multiple discrete tan-brown pin-head papules	RP	Type 2	[53]
Female	29	NR	Dorsal	Hyperpigmentation, diffuse with a symmetrical pattern	NR	Type 2	[61]
Female	30	NR	Dorsal	Hyperpigmented macules	RP	Type 2	[30]
Female	30	Black	Anterial	Multiple hyperpigmented papillae	RP	Type 2	[74]
Female	32	Caucasian	Dorsal	6 mm oval area with brown pigmentation	NR	Type 2	[75]
Female	35	Japanese	Antero-lateral	Hyperpigmented papillae	RP	Type 1	[44]
Female	35	African	Dorsal	Groups of 15 to 20 papillae with a mottled appearance	NR	Type 2	[76]
Female	43	South American	Dorsal	Diffuse and symmetrical pattern of macules	NR	Type 1	
Female	40	Black	Anterial	Multiple hyperpigmented papillae	RP	Type 2	[39]
Female	44	Black	Anterial	Multiple hyperpigmented papillae	RP	Type 2	
Female	44	African	Antero-lateral	NR	NR	Type 2	[77]
Female	45	Hispanic	Lateral	Multiple dark-brown pigmented papules	NR	Type 1	[78]
Female	NR *	NR	Dorsal and lateral	Multiple hyperpigmented papillae and patches	CS	Type 2	[38]
Male	8	Latin America	Anterial and dorsal	Multiple asymptomatic and sharply demarcated hyperpigmented pinhead papules	CS	Type 2	[59]
Male	11	Brazilian	Anterial and lateral	Multiple tiny brown macules	NR	Type 1	[62]
Male	12	NR	Dorsal and lateral	Multiple pigmented papule in a symmetrical pattern	NR	Type 1	[41]
Male	17	Korean	Anterial	Well-demarcated small, black, clustered hyperpigmented papules	NR	Type 1	[79]
Male	26	Taiwanese	Antero-lateral	Multiple tiny brown macules	CS	Type 2	[80]
Male	28	NR	Anterial	Multiple dark-brown macules and dome shaped papules	CS	Type 1	[34]
Male	28	Hispanic	Latero-distal	Multiple dark-brown pigmented papules	RP	NCP	[35]
Male	36	Italian	Dorsal and lateral	Multiple brown papillae	CS	Type 2	[81]
Male	42	Japanese	Lateral	Black pigmented papillae	NR	Type 1	[40]
Male	65	Vietnamese	Dorsal and lateral	NR	NR	Type 2	[82]
Female	21	Javanese	Dorsal and lateral	Multiple brownish-black, diffuse, and asymptomatic macules	CS	Type 2	[60]
Male	22	Javanese	Dorsal	Multiple macules, brownish-black and sharing a clear border	CS	Type 2	
Female Female Female Female Female Female Female Female Female Female Female Female Male Male Male	18 18 22 26 27 29 31 36 39 40 48 51 8 24 52	Mixed ethnicity	Not reported individually in each patient	patient	Not reported individually in each patient	Type 2 Type 2 Type 2 Type 2 Type 2 Type 2 Type 2 Type 1 Type 2 Type 2 Type 2 Type 2 Type 1 Type 2 Type 2	[54]

NR: Not reported; * not mentioned specifically (mentioned as adolescent); CS: Cobblestone appearance; RP: Rose-petal appearance; NCP: No clinical picture.

### 3.4. Complete Blood Cell Count and Blood Chemistry Findings

The following additional investigations were reported in some studies: routine laboratory [53,58], complete blood cell count [36,37,40,41,46,47,49,62,64,69,70,71,79,81,82], blood glucose, vitamin levels, trace element (iron, zinc), serum electrolytes, and blood chemistry (urea, ferritin).

The complete blood cell count was reported in 15 studies, mostly with normal results. Only in three studies were some anomalies detected, including low hemoglobin [41], low leucocyte count [41], higher mean corpuscle volume (MCV) [82], and heterozygosity of hemoglobin [36]. The blood glucose [51,69,71,82], vitamin levels [36,49,51,52,69,82], trace element [36,46,47,62,68,82], electrolytes [37,51,69,71,81], and blood chemistry, including ferritin [49,51,52,69] and urea [37,69,70,71] were reported within normal limits, as well as metabolic panel [46,47,49,62] and some analyses stated as routine laboratory investigations [53,58]. Only one study reported a high level of iron in a Vietnamese male [82].

### 3.5. Liver and Kidney Function Tests

Eleven studies reported liver and renal function tests, or urinalysis [36,37,40,51,68,69,70,71,79,82]. The liver function test was reported normal as well as renal function test, creatinine levels, and urinalysis in nine studies. One study reported a high level of aspartate aminotransferase, alanine aminotransferase, and bilirubin [81], and in other study high levels of anti-smooth muscle antibodies, anti-liver antibodies, and anti-kidney microsomal antibodies were detected [82].

### 3.6. Infection Markers, Endocrine, Auto-Immune, Ophthalmological, and Neurological Findings

Diverse additional investigations were reported, including infection markers (hepatitis [51], syphilis [79], fungal infection [52], and HIV [76]), endocrinological tests [40,79] (synacthenor adrenal cortex function [64], hyperinsulinism [36], and thyroid [47,49,82]) and auto-immune (anti-nuclear antibodies [46,62,82]) tests, ophthalmological and neurological examinations [40,79]. All studies reported that the infection markers were negative, with the exception of one study in which the HIV test was positive [76].

### 3.7. Additional Findings

No antecedent of medication intake was reported in 14 studies [31,39,41,46,50,61,67,68,72,73,74,75,76,80]. Similarly, history of a relative with a similar condition [34,38,41,42,46,49,51,57,61,62,68,70,72], social habits (smoking and tobacco) [31,67,73,81], or relevant medical history [31,38,43,46,48,49,50,57,58,59,65,68,70,75,78,81] were not found in 26 studies. Only one study reported that the PFP became more noticeable during pregnancy in a Caucasian 32-year-old female [75], and one 30-year-old female patient had concomitant Dowling–Degos disease [30].

Five studies reported that PFP were found in patients with active medication. Medications included oral iron; II-glycine-sulfate [47]; tenofovir, emtricitabine, and nevirapine [76]; paucibacillary [39]; interferon and ribavirin [82]; and meloxicam [60]. Two patients were reported with higher body mass index (obesity) [36,60]. Two patients had PFP similar to their mother [55,56], the first one with Indian ethnicity and the other with no race reported. Three patients presented with iron deficiency anemia [47], condyle arthritis [60], or recurrent aphthous ulceration [82].

### 3.8. Histopathology

The histopathological descriptions of PFP revealed the following general characteristics: (i) hyperpigmentation of melanocytes with brown melanin of the basal cell layers of epithelium; (ii) melanophages in the lamina propria, sub-epithelial or sub-mucosal connective tissue; (iii) scant lymphocytic infiltrate in the lamina propria; (iv) chromatophores in the sub-epithelial area and surrounding the blood vessels of the fungiform papillae; and finally, (v) dilated vascular spaces (Table 2).

### 3.9. Immunohistochemistry

Immunohistochemical studies showed an immune-reactive for CD3+ T lymphocytes, S-100 and Sox10 in the intraepithelial melanocytes and non-immuno-reactive melan-A (Table 3).

### 3.10. Other Features

Using specific staining, two studies showed the presence of melanin with Fontana–Masson staining. PFP were not linked with the presence of iron or hemosiderin with Berlin blue and Perl’s staining (Table 3).

## 4. Discussion

The tongue is the largest organ of the oral cavity, and its surface is studded with four types of papillae: filiform, fungiform, foliate, and vallate papillae. Lingual papillae are thought to increase the surface area of the tongue, serve as bearers of taste buds, and also to increase the area of contact and friction between the tongue and food [45]. The fungiform papillae are club shaped projections generally found scattered on the tip and sides of the tongue. They are, in health, pink to red in colour as the rest of the lingual mucosa, and usually do not stand out as discrete organelles. However, in some people, the fungiform papillae are pigmented and can be readily seen as discrete projections, even by naked eye examination. In most of the reports of the current review PFP appeared as dark-brown, blue-grey, or bluish-black papillae.

A number of hypotheses have been proposed for the origin of PFP. Some contend that it is due to the transfer of darker melanocytes in the basal layer of the epithelium to the superficial keratinocytes via membrane-bound organelles called melanosomes [83]. The melanin is likely to be the darker variant eumelanin found in black ethnicity, as opposed to the lighter variant pheomelanin seen in Asian ethnicity [84]. The melanin was later confirmed in PFP, and not related to the presence of iron and hemosiderin [43,44].

**Figure 2:**
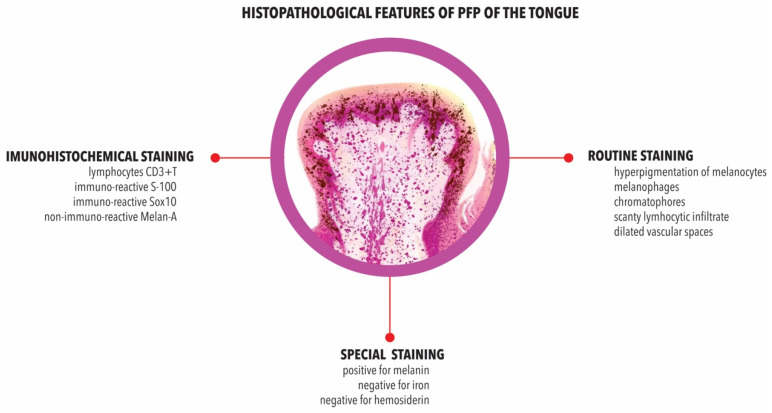
Schematic illustration of histopathological features of PFP of the tongue.

On histological examination of the papillary epithelium, PFP appear to be due to the hyperpigmentation of papillary keratinocytes [34,43,73,80], usually bearing a profusion of melanocytes containing melanin granules in the basal cell layer of the epithelium [64,73]. Other features include a mild to moderate lymphocytic infiltrate of the lamina propria [54,72], and chromatophores in the sub-epithelial area, and around the blood vessels of the papillae [33]. Many authors have observed melanophages containing melanin granules in the lamina propria [34,35,40,51,67,69,70,73,79], the connective tissue [64], subepithelial tissue [54,72], adjacent filiform papillae [75], and the submucosa [65,73] of the fungiform papillae (Figure 2).

Melanophages—macrophages that contain melanin—are a common feature in inflammatory and non-inflammatory pigmentation [85]. Melanophages are large cells with indistinct cytoplasmic boundaries, usually located around or near superficial dermal vessels [73]. Another histological feature described by some authors in PFP is the presence of so-called ‘chromatophores’, a disputed, and confusing term used by Holzwanger [33,71] and Koplon [71] in the older literature, referring to melanocytes, or perhaps more probably to melanophages (pigment carrying cells) as mentioned by Steigmann [86]. The presence of melanin was later confirmed with Fontana–Masson staining [44].

The presence of a lymphocytic infiltrate reported in some studies, could be considered as a sign of an inflammatory response associated with the genesis of PFP. In an immunohistochemical study, Ghigliotti et al. revealed that the lymphocytes in PFP were mainly CD3 + T lymphocytes [72]. The localised traumatic event or mechanical injury provokes inflammatory responses. The inflammatory responses cause inflammatory cytokine production from keratinocytes as well as from fibroblasts, which in turn stimulate melanocytes, often resulting in pigmentation. In some research, it has been mentioned that several cytokines such as interleukin, tumor necrosis factor (TNF), and prostaglandin E (PGE) modulate the proliferation and differentiation of human melanocytes to pigmentation [87]. The presence of the latter lymphocytic infiltrate, together with the melanophages, implies the possibility that the pigmentation is due to an inflammatory process, provoked most likely by a localised traumatic event (Figure 2).

Not surprisingly, some authors have investigated whether PFP is a precursor of melanoma. To our knowledge, no malignant transformation has been reported from PFP. Only in two studies were immunohistochemical staining for melanotic markers done, detecting Sox10 [43] and S-100 [75] expressions, but they were non-reactive for melan-A [75].

On further detailed review of the reports, we noted that a number of investigators have used dermoscopy to evaluate the nature of PFP. This technique increases the sensitivity and specificity of the clinical examination, and almost eliminates biopsy procedures and by extension reduces patient discomfort. Nevertheless, dermoscopy is infrequently used in dentistry [88]. A majority of clinicians in our review used dermoscopy, but in combination with biopsy examination, which seems to be a waste of resources [34,35,64,65,67,72,80].

**Figure 3:**
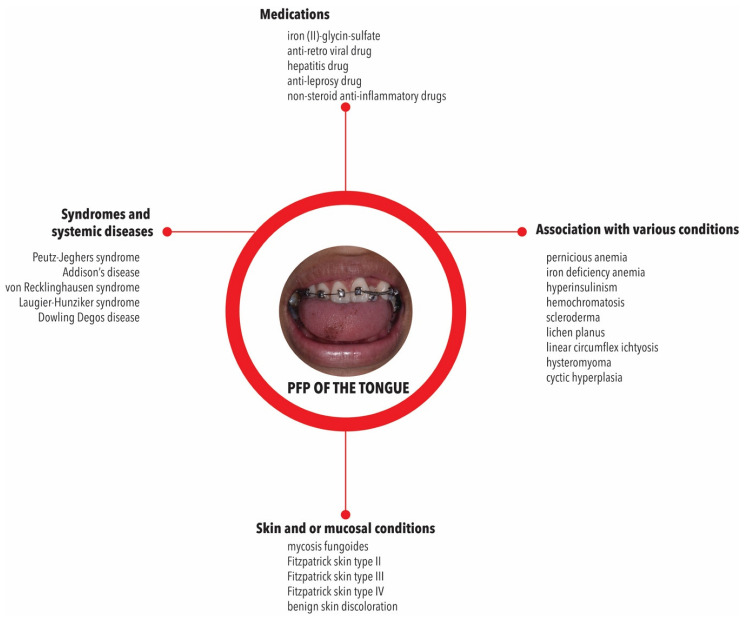
Associations described for PFP with syndromes, systemic conditions, medications, and other related conditions.

Some of the reviewed reports showed that PFP were associated with other skin and/or mucosal conditions, including mycosis fungoides [34,35], Fitzpatrick skin type II [42], Fitzpatrick skin type III [44,54], Fitzpatrick skin type IV [46,47,48,49,50,51,52,53,54], yellowish discolourations in sclera and conjunctival mucosa [41], and benign skin discolourations [36,37,38,39,40]. Nonspecific observations have been made in this context, as a few cases were associated with light brown skin—i.e., Fitzpatrick skin type IV [46,48,50]—or less frequently with fair skin and blue eyes-skin type II [42]. However, many reports did not mention the skin colour [60,79,89] (Figure 3).

PFP were also described in association with other syndromes and systemic diseases including Peutz–Jeghers syndrome [35,40,51,64,67,72,73], Addison’s disease [35,40,51,64,67,72,73], von Recklinghausen syndrome [51,72], and Laugier–Hunziker syndrome [64,73,80] (Figure 3). Two cases reported that the PFP were inherited, because this condition was found in the mother and her children [55,56] as also in a case of Dowling Degos disease (EDD) [30].

The role of external factors in the origin of PFP was also analysed. Medication intake [31,39,41,46,50,61,67,68,72,73,74,75,76,80], family history [34,38,41,42,46,49,51,57,61,62,68,70,72], medical history [31,38,43,46,48,49,50,57,58,59,65,68,70,75,78,81], smoking [31,67], and tobacco use [67,73] did not appear to be related. However, in some cases, PFP were found to be related with the medication, such as iron (II)-glycinee-sulfate [47], and anti-retro viral drug [76], hepatitis drug [82], anti-leprosy drug [66], and non-steroid anti-inflammatory drugs [60]. They have also been found in HIV infected patients [76]. Specific pigmentation of the papillae related to human immunodeficiency virus (HIV) infection [8] and medication-induced [90,91] are common causes of mucosal pigmentation and are not directly involved in PFP pathogenesis.

In order to ascertain the aetiology of PFP and to rule out systemic diseases, numerous laboratory analyses have been conducted, including complete blood cell count [37,40,46,47,49,62,64,69,70,71,79,81], blood glucose [69,71,82], or fasting blood glucose level [51], liver function tests [36,37,51,69,79], renal or kidney function tests [36,68,79,82], urinalysis [40,70,71,79], adrenal cortex function (synacthen) tests [64], electrolyte levels [37,51,69,70,81], vitamin B12 [49,51,52,69], vitamin D [36], folate [82], trace element [36,46,47,62,68,82], ferritin [49,51,52], and urea [37,69,71]. The infection markers, as hepatitis markers [51], fungal [52], VDRL [79], the endocrinological [36,40,47,49,64,79,82], and anti-nuclear antibody levels [46,62,82] were assessed. The results of the foregoing tests as well as ophthalmological and neurological tests conducted by some [40,79], were negative, and did not reveal an oral-systemic connection with the origin of PFP. Only three cases showed low hemoglobin and leucocytes counts [41] and high MCV counts [82], heterozygocity of HBs [36] was noted amongst the 69 studies reviewed. These data point to the fact that PFP is a normal physiological condition with an aberration of the focal melanocyte function of the fungiform papillae.

On the other hand, PFP have been associated with various systemic conditions, such as pernicious anemia [41,51,64,72,73], iron deficiency anemia [41], hemochromatosis [41,51,64,72,73], scleroderma [41,73,79], lichen planus [34,41], linear circumflex ichthyosis [34,41], hysteromyoma [34], and cystic hyperplasia [34]. Some patients presented lower hemoglobin and total leukocyte counts [41], lower serum iron, 25 OH vitamin D, zinc, and hyperinsulinism [36], higher levels of aspartate aminotransferase, alanine aminotransferase and bilirubinemia [81]. These conditions appear to be mere associations with PFP and not the direct cause, as far as we gathered from our meta-analysis.

**Figure 4:**
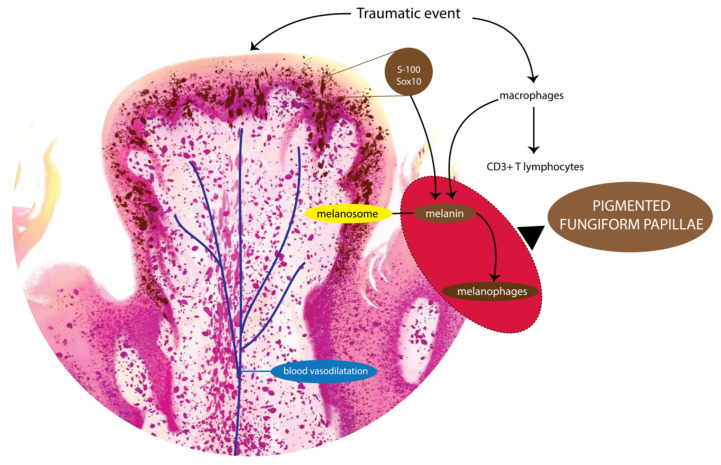
Proposed model for the pathogenesis of PFP.

Based on the current study findings and interpretation, it may be speculated that PFP could arise due to injury and disruption of the vascular architecture of the fungiform papillae during masticatory traumatic events. This would cause the extravasation of blood and a subsequent influx of inflammatory infiltrate of macrophages and lymphocytes into the papillary tissue [64]. Sugiyama et al. have postulated that the inflammatory process then causes an increase in melanin synthesis in the lamina propria of the fungiform papillae leading to the hyperpigmentation [64]. However, this fact does not explain why only the fungiform papillae—and not the other papillary variants of the lingual mucosa—are affected by such events. Moreover, if traumatic events were the main cause acting in the origin of PFP, it would be expected to occur more frequently and at any age. Its description, affecting several and different ethnicities as we observed in our study, does not add further information about a possible predisposition in that sense, although an apparent predominance among Asian versus White people was noted. These findings contrast with those of Holzwanger [33], who stated that PFP is a relatively common variant of oral pigmentation which, although more prevalent in Negroes, it is seen in other heavily pigmented races as well. Therefore, the reason/s for the focal pigmentation of fungiform papillae is/are yet speculative, and remain to be clarified (Figure 4).

The major limitation of this study is the absence of systematic evaluations of PFP, despite the plethora of case reports which are mainly anecdotal in nature. This leads to difficulties in systematic data collection. Furthermore, none of the case reports were followed up, and it is unclear whether the PFP is a transient phenomenon or lasts for prolonged periods. Hence prospective studies are needed.

## 5. Conclusions

Our findings, taken together, clearly indicate that PFP is a normal physiological condition with an aberration of the focal melanocyte function of the fungiform papillae of the tongue. Some reports indicate that the condition could be associated with several systemic conditions and syndromes but, clearly, not caused by them. The inflammatory infiltrate seen in some papillary lesions are likely to be due to masticatory trauma. Yet, the true reasons for the discolouration of fungiform papillae are as of yet elusive.

## Figures and Tables

**Table 2:** Histopathological findings in the reported cases of selected studies.

Gender	Ages	Ethnicity	Histopathological Findings	Ref
Female	9	Japanese	- Melanocytes containing brown melanin granules were present along the basal cell layer of the epithelium - Melanophages containing melanin granules were observed within the connective tissue	[64]
Female	9	NR	- Basal cell layer of keratinocytes with a high content of brown pigment - Accumulation of melanophages - Moderate lymphocytic infiltration	[43]
Female	12	Asian	Melanophages in the submucosa	[65]
Female	18	Black	Chromatophores in the subepidermal area and around the blood vessels within the fungiform papillae	[33]
Female	18	NR	Melanophages were found in the lamina propria of the papillae	[51]
Female	20	NR	Melanophages in the lamina propria	[67]
Female	24	Hispanic	Melanophages in the lamina propria	[69]
Female	25	Korean	Melanophages in the upper lamina propria	[70]
Female	33	Korean	Melanophages in the upper lamina propria	
Female	26	Black	- Chromatophores containing melanin in the underlying dermis	[71]
Female	27	Italian	- Melanophages in the sub-epithelium - Slight lymphocytic infiltrate in the lamina propria	[72]
Female	28	Haitian	- Stratified squamous epithelium overlying a loose connective tissue core - Melanocytes and keratinocytes containing brown melanin granules were present along the base of the epithelium - Melanophages with coarser melanin granules within the connective tissue core or lamina propria	[73]
Female	32	Caucasian	Melanophages were prominent in the fungiform and adjacent filiform papillae	[75]
Female	35	Japanese	- Increased basal pigment and melanophages in the lamina propria within the fungiform papillae - No significant melanocytic hyperplasia was noted - Cell atypia not present	[44]
Female	45	Hispanic	Melanin deposits in the epithelium	[78]
Male	12	NR	Melanophages in the subepidermal area	[41]
Male	17	Korean	Melanophages in the upper lamina propria without significant inflammation	[79]
Male	26	Taiwanese	Pigmented basal keratinocytes and melanophages in the upper lamina propria	[80]
Male	28	NR	- Melanophages in the lamina propria - Hyperpigmentation of basal keratinocytes	[34]
Male	28	Hispanic	Melanophages within the lamina propria	[35]
Male	42	Japanese	Melanophages in the upper lamina propria without significant inflammation	[40]
Female Female Female Female Female Female Female Female Female Female Female Female Male Male Male	18 18 22 26 27 29 31 36 39 40 48 51 8 24 52	Mixed ethnicity	- Subepithelial melanophages - Mild lymphocytic infiltrate in the superficial area - Dilated vascular spaces	[54]

NR: not reported.

**Table 3:** Histological findings based on staining of PFP.

Gender	Ages	Ethnicity	Routine Staining		Special Staining		Immunohistochemical Staining		Ref.
			Marker	Staining	Marker	Staining	Marker	Staining	
Female	9	NR	Brown pigment in the basal layer of keratinocytes	HE	Iron and hemosiderin not detected	Perl’s	Immuno-reactive for Sox-10	IHC	[43]
Female	27	Italian	- Melanophages in the sub-epithelial - Slight lymphocytic infiltrate in the lamina propria	HE	-	-	Immuno-reactive of CD3+ T lymphocytes	IHC	[72]
Female	32	Caucasian	The stroma supporting the fungiform and adjacent filiform papillae showed prominent melanophages	HE	-	-	- No immuno-reactive for Melan-A in the melanocytes - Immuno-reactive for S-100 protein in the melanocytes	IHC	[75]
Female	35	Japanese	- Increased basal pigment in the lamina propria - Increased melanophages in the lamina propria - No significant melanocytic hyperplasia was noted - No cells showed atypia	HE	Melanin was detected	Fontana–Masson	-	-	[44]
					Iron and hemosiderin not detected	Berlin blue	-	-	

NR: Not reported. HE: Hematoxylin-eosin. IHC: Immunohistochemistry.

## Data Availability

Not applicable.
